# Pro-invasive Effect of Proto-oncogene PBF Is Modulated by an Interaction with Cortactin

**DOI:** 10.1210/jc.2016-1932

**Published:** 2016-09-07

**Authors:** Rachel J. Watkins, Waraporn Imruetaicharoenchoke, Martin L. Read, Neil Sharma, Vikki L. Poole, Erica Gentilin, Sukhchain Bansal, Emy Bosseboeuf, Rachel Fletcher, Hannah R. Nieto, Ujjal Mallick, Allan Hackshaw, Hisham Mehanna, Kristien Boelaert, Vicki E. Smith, Christopher J. McCabe

**Affiliations:** Institute of Metabolism and Systems Research (R.J.W., W.I., M.L.R., N.S., V.L.P., S.B., R.F., H.R.N., K.B., V.E.S., C.J.M.), University of Birmingham, Birmingham B15 2TT, United Kingdom; Department of Surgery, Faculty of Medicine (W.I.), Siriraj Hospital, Mahidol University, Bangkok 10700, Thailand; Section of Endocrinology and Internal Medicine (E.G.), University of Ferrara, 44121 Ferrara, Italy; STIM Laboratory (E.B.), University of Poitiers, 86073 Poitiers Cedex 9, France; Northern Centre for Cancer Care (U.M.), Freeman Hospital, Newcastle upon Tyne NE7 7DN, United Kingdom; Cancer Research United Kingdom & UCL Cancer Trials Centre (A.H.), University College London, London WC1E 6BT, United Kingdom; and Institute of Cancer and Genomic Sciences (H.M.), University of Birmingham, Birmingham B15 2TT, United Kingdom

## Abstract

**Context:**

Metastatic disease is responsible for the majority of endocrine cancer deaths. New therapeutic targets are urgently needed to improve patient survival rates.

**Objective:**

The proto-oncogene PTTG1-binding factor (PBF/PTTG1IP) is overexpressed in multiple endocrine cancers and circumstantially associated with tumor aggressiveness. This study aimed to understand the role of PBF in tumor cell invasion and identify possible routes to inhibit its action.

**Design, Setting, Patients, and Interventions:**

Thyroid, breast, and colorectal cells were transfected with PBF and cultured for in vitro analysis. PBF and cortactin (CTTN) expression was determined in differentiated thyroid cancer and The Cancer Genome Atlas RNA-seq data.

**Primary Outcome Measure:**

Pro-invasive effects of PBF were evaluated by 2D Boyden chamber, 3D organotypic, and proximity ligation assays.

**Results:**

Our study identified that PBF and CTTN physically interact and co-localize, and that this occurs at the cell periphery, particularly at the leading edge of migrating cancer cells. Critically, PBF induces potent cellular invasion and migration in thyroid and breast cancer cells, which is entirely abrogated in the absence of CTTN. Importantly, we found that CTTN is over-expressed in differentiated thyroid cancer, particularly in patients with regional lymph node metastasis, which significantly correlates with elevated PBF expression. Mutation of PBF (Y174A) or pharmacological intervention modulates the PBF: CTTN interaction and attenuates the invasive properties of cancer cells.

**Conclusion:**

Our results demonstrate a unique role for PBF in regulating CTTN function to promote endocrine cell invasion and migration, as well as identify a new targetable interaction to block tumor cell movement.

## Abstract (precis)

This study identifies a novel interaction between proto-oncogene PBF and cortactin which opens up new therapeutic avenues for blocking tumour cell movement in endocrine cancer.

New cancer targets, which abrogate the movement of tumor cells, will be critical to improving future cancer survival rates worldwide (1). Of central importance in attaining this goal is to better understand how tumors exhibit uncontrolled and invasive cellular growth, which is modulated through the interaction of malignant cells with the extracellular matrix, the vasculature, and the immune system (2, 3). Considerable attention has focused on the regulation of actin assembly at the leading edge of malignant cells, a process integral to the formation of protrusive structures such as invadopodia (4). It is known, for instance, that a multitude of diverse signaling molecules such as RhoA can control actin cytoskeletal dynamics in migrating cells (5). However, factors that influence actin polymerization in the tumor environment are still poorly understood.

Cortactin (CTTN) is a scaffold protein active predominantly at the periphery of cells which promotes actin polymerization by binding and activating the Arp2/3 complex (6), as well as inhibiting debranching of actin networks (7). In this way, CTTN is able to exert a potent influence upon cell movement and invasion (8–10). Indeed, overexpression of CTTN is associated with increased cellular motility and invasiveness in multiple cancer settings (8, 9). CTTN function is multifaceted and has been linked to numerous processes, including vesicular trafficking (8, 11), focal adhesion dynamics (12) and extracellular matrix secretion (13). These studies emphasize the importance of CTTN as a key player in aggressive cancers but further work is required to establish the precise role of CTTN in different tumor settings.

Modulators of the complex processes by which cells invade, migrate, and metastasize continue to be identified (14). We initially characterized a potential role for the proto-oncogene PTTG1-binding factor (PBF/PTTG1IP) in breast cancer cell invasion in vitro (15). Subsequently, three different studies have circumstantially linked PBF to the process of tumor invasion and metastasis in vivo. First, high PBF expression was correlated with distant metastasis at diagnosis, tumor node metastasis (TNM) stage and disease-specific survival in a large series of papillary thyroid cancers (16). Secondly, high PBF promoter activity was associated with poorer clinical outcome and increased metastatic risk in breast cancer (17). Thirdly, colorectal tumors with higher PBF protein expression demonstrated greater vascular invasion (18). Thus, it is possible that PBF is integral to tumor cell invasion in vivo.

PBF was first identified as a type 1A integral membrane protein (IMP) (19). IMPs with cytosolic domains can act as anchors for cytoskeletal proteins or be involved with intracellular signaling. Its best described role however lies in binding the sodium iodide symporter NIS and regulating its subcellular localization within thyroid cells (20, 21). PBF also interacts with the tumor suppressor p53 (18, 22) and the protein kinase Src (23). In particular, PBF is a phospho-protein and interaction with Src elicits phosphorylation at tyrosine (Y) residue 174. This residue has a potential functional duality as it forms the first residue of a consensus YXXΦ internalization motif. However, although pY174 is enriched at the plasma membrane (PM) (23), the precise relationship between PBF phosphorylation and intracellular trafficking of its binding partners requires further investigation.

Given the emerging roles of PBF in cancer, particularly as a proto-oncogene, here we studied its role in migration and metastasis. We conducted mass spectrometry in thyroid cancer cells to map the full range of interactions with PBF and identified CTTN as our top hit. As CTTN has a well described role in cell migration and tumor metastasis, we assessed whether the interaction between CTTN and PBF was functional. Extensive data are presented validating and delineating the interaction between the two proteins. Thus we identify a new functional binding partner of CTTN, and propose a novel mechanism whereby PBF binds CTTN close to the PM to facilitate cellular movement. Modulation of PBF activity may therefore represent a promising new target for addressing tumor cell invasion and migration.

## Materials and Methods

### Human tissue and cell culture

Human thyroid samples were obtained with local ethics committee approval and informed patient consent. Breast (MCF-7, MDA-MB-231) and thyroid (TPC-1, SW1736) cell lines were maintained in RPMI 1640 (Life Technologies). HCT116 cells were maintained in McCoy's 5A (Life Technologies), whereas HeLa and NIH3T3 cells were maintained in DMEM (Sigma-Aldrich). All media was supplemented with 10% fetal bovine serum, penicillin (10^5^ U/L), and streptomycin (100 mg/L). All cell lines were obtained from the European Collection of Authenticated Cell Cultures, except TPC-1 and SW1736, which were kindly provided by Dr Rebecca Schweppe (University of Colorado). Cells were cultured as recommended at low passage and authenticated by short tandem repeat analysis (DNA Diagnostics Centre). Stable cell lines were constructed as described previously (36). Further information on cell lines (Supplemental Table 1) and a summary of endogenous PBF and CTTN levels are provided (see Supplemental Figure 13).

### Datasets

Normalized gene expression data generated using the Illuminia RNA-seq platform and clinical information was downloaded from cBioPortal (37, 38). Gene expression values were transformed as X = log_2_(X + 1) where X represents the normalized fragments per kilobase transcript per million mapped reads (FPKM) values. For matched normal (N) and tumor (C) pairs relative fold-changes (FC) were transformed as log_2_FC = log_2_(C) − log_2_(N) where C and N represent normalized FPKM values. Transcriptome datasets were selected for tumors with TNM staging of T1–3N0 (nonmetastatic) or T1–3N1 (lymph node metastasis). Transcriptomic and clinical information was analyzed for 326 patients with thyroid cancer.

### Nucleic acids and transfection

Plasmids containing PBF cDNA with a hemagglutinin (HA) tag have been described (37). The QuikChange Site-Directed Mutagenesis Kit (Stratagene) was used to generate Y174A PBF-HA. Plasmids expressing human Myc-CTTN (No. RC223259) and GFP-PBF (No. RG202109) were purchased from Origene (Rockwell). CTTN siRNAs (s4666 and s4667) and control siRNA (AM4635) were purchased from Invitrogen. Plasmid DNA and siRNA transfections were performed with TransIT-LT1 (Geneflow) and siPORT (Invitrogen) using standard protocols.

### Western blotting and antibodies

Western blot analyses was performed as described previously (21, 31). Blots were probed with specific antibodies against PBF [Eurogentec; (15, 21)], pY174 PBF [CovalAb (23)], HA (16B12; Covance Research Products), HA (sc-805; Santa Cruz), Myc (No. 2276; Cell Signaling Technology), CTTN (AB3887; Merck Millipore), pCTTN (Tyr421) (No. 4569; Cell Signaling Technology), and β-actin (AC-15; Sigma-Aldrich). AlexFluor-conjugated antibodies (488, 555, 594 and 647) were purchased from Molecular Probes.

### Immunostaining and proximity ligation assay

Immunofluorescence was performed as described previously (21). A Zeiss confocal LSM 510 microscope was used to perform confocal microscopy. Images were processed with ImageJ (http://imagej.net/Coloc_2) to determine Pearson's colocalization coefficients (39). Epifluorescent microscopy was performed on a Zeiss Axioplan fluorescent microscope. Human thyroid tissue was immunostained using the Novolink polymer detection kit (Leica) as per manufacturer's instructions. Slides were viewed under a light microscope (Zeiss) and images captured using Axiovision software. The Duolink in situ proximity ligation assay (PLA) was performed according to manufacturer's instructions (Olink Bioscience).

### Invasion assays and live cell imaging

2D Boyden chamber invasion assays were performed according to manufacturer's instructions using BioCoat growth factor reduced matrigel invasion chambers (BD Biosciences). 3D organotypic invasion assays were performed using standard protocols with MDA-MB-231 cells and cancer-associated fibroblasts. Invasion parameters were calculated as described previously (40). Classical scratch wound healing assays were performed using standard protocols and live cell images taken with a Nikon A1R Inverted Confocal/TIRF microscope.

### Mass spectrometry

PBF-interacting proteins were isolated by coimmunoprecipitation (anti-mHA antibody) and separated by SDS-PAGE. Following destaining, proteins were reduced, alkylated, and trypsinized before undergoing HPLC and AmaZon ETD ion trap and tandem mass spectrometry (Bruker Daltronics). Mass spectrographs were analyzed via the Mascot search engine (Matrix Science) and ProteinScape (Bruker). Multiple runs were performed to identify consistent interactors.

### RNA extraction and qRT-PCR

Total RNA was extracted from human thyroid tissue using the RNeasy FFPE kit (QIAGEN) as per manufacturer's instructions. Expression of specific mRNAs was determined on a 7500 Real-time PCR system (Applied Biosystems) using the QuantiTect Probe RT-PCR kit (QIAGEN). Relative expression was determined using the 2^−ΔΔCt^ method. TaqMan assays are described (Supplemental Table 3).

### Statistical analysis

All results were obtained from triplicate experiments unless otherwise indicated. Data were analyzed using SigmaStat (SPSS) and displayed as mean ± SEM. Statistical analyses were performed with the Student *t* test or the nonparametric Mann-Whitney *U* test. We used Spearman's rank-based correlation to determine associations between TCGA datasets. Significance was taken as *P* < .05.

## Results

### PBF induces cellular invasion and binds CTTN

**Figure 1.**
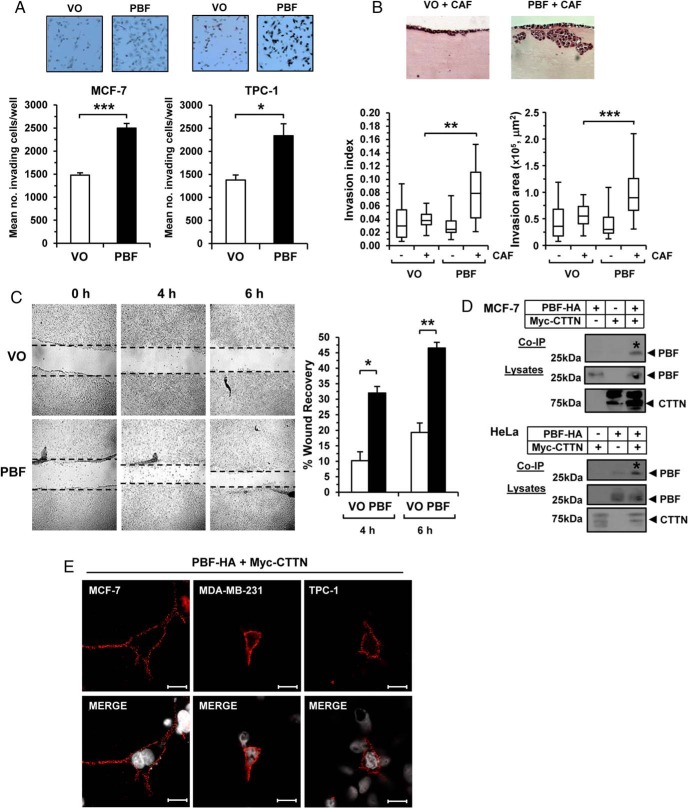
Proinvasive effect of PBF and specific binding to CTTN. A, Representative 2D Boyden cell invasion assays from three independent experiments showing increased invasion of MCF-7 and TPC-1 cells transfected with PBF compared with VO. Results are mean ± SEM. Representative photomicrographs shown above. B, Representative 3D organotypic invasion assays in MDA-MB-231 cells transfected with VO or PBF. Box-whisper plots show quantification of invasion index and area of MDA-MB-231 cells in the presence (+) or absence (−) of cancer-associated fibroblasts. Statistics analyzed using Mann-Whitney *U* test. C, Representative images of scratch wound assay from three independent experiments in NIH3T3 cells stably transfected with VO or PBF at 4 and 6 h postrecovery with percent wound recovery shown (graph). Results are mean ± SEM. *, *P* < .05; **, *P* < .01; ***, *P* < .001. D, Representative Co-IP assays from three independent experiments showing specific interaction (*) between myc-tagged CTTN and HA-tagged PBF in MCF-7 and HeLa cells. E, Proximity ligation assays (PLA) assays showing specific interaction between PBF-HA and myc-CTTN in MCF-7, MDA-MB-231, and TPC-1 cells. Red fluorescent spots indicate specific interactions. White indicates DAPI nuclear staining. Magnification, 63×. Scale bars, 10 μM.

The precise role of PBF in tumor cell invasion remains poorly understood in breast, thyroid, and colorectal cancer cells (15–18). We therefore sought to investigate the ability of PBF to induce invasion across a panel of cancer cells (Supplemental Table 1). The potent proinvasive effect of PBF overexpression was evident in 2D assays in MCF-7, TPC-1, and HCT116 cell lines (Figure 1A and Supplemental Figure 1A; *P* < .05). We further performed 3D organotypic invasion assays in MDA-MB-231 breast cells and showed that PBF is proinvasive in vitro with significantly increased invasion parameters (Figure 1B and Supplemental Figure 1B; *P* < .01). Cell invasion is related to and encompasses cell migration. We therefore next evaluated cell migration to determine whether elevated levels of PBF are promigratory. A stable murine NIH3T3 cell line overexpressing PBF (>24-fold increase in PBF mRNA) demonstrated that PBF induced significant wound healing compared with controls at both 4 and 6 hours postrecovery (Figure 1C).

Having established that PBF exhibits a broad and consistent role in promoting cell invasion, we next performed mass spectrometry in thyroidal TPC-1 and K1 cell lines to identify possible binding partners of PBF with roles in cell motility. From our in vitro studies we identified CTTN, which was the top hit across all runs (12 peptide hits; average score per peptide = 40.0; n = 4 runs; Supplemental Table 2). Given CTTN's pivotal role in cell migration and invasion, we appraised the mass spectrometry data using coimmunoprecipitation (co-IP) assays in MCF-7 and HeLa cells. These confirmed a specific interaction between PBF and CTTN in vitro (Figure 1D). We challenged our findings with the separate methodology of proximity ligation assays (PLAs); further demonstrating specific binding between CTTN and PBF predominantly toward the periphery of cells (Figure 1E). No specific interactions were apparent in control PLA assays (Supplemental Figure 1C).

### CTTN and PBF colocalize during cell migration

**Figure 2.**
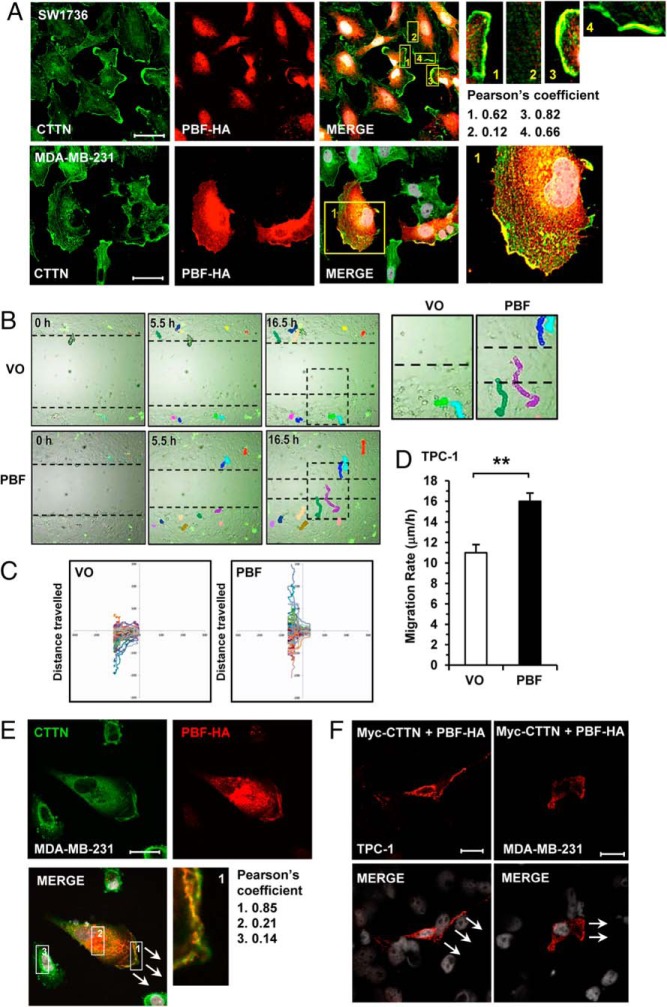
PBF and CTTN colocalize at the leading edge in actively migrating cells. A, Representative confocal images showing endogenous CTTN (green), exogenous PBF (red) and colocalization (yellow) in SW1736 and MDA-MB-231 cells. Framed areas in merge images are magnified in right panels and Pearson's colocalization coefficients indicated. Magnification, 100×. Scale bars, 20 μM. B, Representative image of live cell migration assay in TPC-1 cells transfected with either GFP-tagged PBF or GFP-VO and tracked over 16.5 h. Photomicrographs show the tracks of individual cells over time. Boxed areas are magnified (right panels). C, Representation of individual tracks within one movie illustrate the further distances traveled by PBF-transfected cells. D, Quantification of mean cell migration rate (μm/h) of TPC-1 cells (n = 79 VO vs n = 63 PBF cells). E, Representative confocal images showing endogenous CTTN (green), exogenous PBF (red) and colocalization (yellow) in MDA-MB-231 cells. Scratch wound migration assays were halted at 4 h. White arrows indicate direction of cell movement. Magnified image from framed area in merge shown in lower right panel and Pearson's colocalization coefficients indicated. F, PLA assays in TPC-1 and MDA-MB-231 cells halted during scratch wound assays showing specific binding between CTTN and PBF (red dots), predominately at the leading edge of migrating cells. Arrows indicate direction of wound healing. White indicates DAPI nuclear stain. Magnification, 63×. Scale bars, 10 μM.

In basal conditions inactive CTTN diffuses throughout the cytoplasm. However, in migrating cells CTTN localizes to the cell cortex to mediate actin filament assembly and promote protrusion formation at the leading edge (10). We next examined CTTN and PBF by immunofluorescence in thyroid SW1736 cells, which revealed CTTN expression predominantly within intracellular vesicles and at the PM (Figure 2A and Supplemental Figure 2). PBF expression was mainly vesicular, and partially overlapped with CTTN, although not consistently within the same vesicular structures. However, colocalization was pronounced at discrete PM regions where CTTN expression was concentrated (Figure 2A and Supplemental Figure 2). Confocal image analysis confirmed significant overlap at intense ‘hot spots’ of PBF:CTTN colocalization at the PM (Figure 2A and Supplemental Figure 2B; see Pearson's coefficient). Together these results demonstrate that CTTN colocalizes with PBF predominately at the PM.

To further investigate the ability of PBF to promote cell migration we performed live cell migration assays in TPC-1 cells transfected with GFP-tagged PBF. Our results demonstrated an overall induction in cellular migration of approximately 40% in the presence of PBF (Figure 2, B–D; *P* < .01). We next determined the colocalization of CTTN and PBF during the process of cellular migration in scratch-wound assays. Our results showed that both proteins had significant colocalization at the leading edge of actively migrating MDA-MB-231 cells (Figure 2E and Supplemental Figure 3A). Again, to determine whether such colocalization was indicative of specific binding, we carried out PLA assays, which revealed that as TPC-1 and MDA-MB-231 cells migrate they are characterized by CTTN and PBF binding, particularly at the leading edge (Figure 2F and Supplemental Figure 3B). Thus, CTTN binds PBF in vitro, a process that occurs in those cells actively undergoing migration.

### CTTN is elevated in differentiated thyroid cancer and correlates with PBF

**Figure 3.**
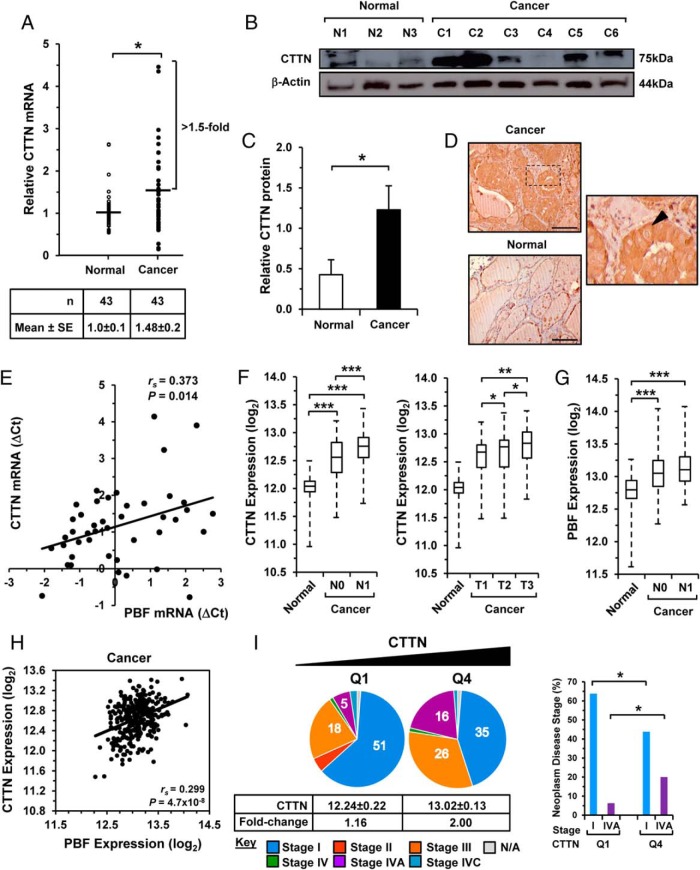
Elevated CTTN expression in thyroid cancer. A, Quantification of CTTN mRNA expression in differentiated thyroid cancer (DTC) relative to matched normal tissue (n = 43). Data represented as a scatter plot. Results are mean ± SEM. *, *P* < .05. B and C, Western blot analysis of CTTN expression in DTC (C1–6) and normal tissue (N1–3) with quantification of relative protein levels (C). Results are mean ± SEM. *, *P* < .05. D, Representative images of CTTN immunostaining in DTC and normal thyroid (20× magnification). CTTN immunostaining was generally cytoplasmic (black arrowhead, high power insert). Scale bars, 100 μM. E, Scatterplot showing a significant correlation between CTTN and PBF mRNA (ΔCT values) in DTC. *P* = .014; *r*_s_ = 0.373; n = 43 (Spearman rank correlation). F, G, CTTN and PBF expression (TCGA) in nonmetastatic (N0; n = 121) and metastatic (N1; n = 205) PTC (left) vs normal thyroid (n = 59), as well as in PTC of different tumor size T1–T3 (right). Data represented in box-whisper plots. *, *P* < .05; **, *P* < .01; ***, *P* < .001 (Mann-Whitney *U* test). H, Scatter plot showing correlation between CTTN and PBF expression (TCGA) in PTC (*P* = 4.7×10^−8^; *r*_s_ = 0.299; n = 326; Spearman rank correlation). I, Association of high (Q4) and low (Q1) CTTN expression with neoplasm staging in PTC (n = 80–82 per quartile). *, *P* < .05.

Although CTTN expression has previously been investigated in breast cancer (24), it has not been addressed in differentiated thyroid cancer (DTC). In a series of matched tumor and normal DTC specimens, CTTN mRNA showed a significant 1.5-fold induction in tumors compared with matched normal thyroid (Figure 3A; *P* < .05; n = 43). CTTN expression was also increased in additional DTC specimens (Figure 3B), with a significant induction in CTTN protein evident in thyroid tumors (Figure 3C; >2.5-fold induction). CTTN was also abundant in DTC as determined through immunohistochemistry (Figure 3D), seeming to be generally cytoplasmic within thyroid epithelial cells.

We next determined the association between CTTN and PBF mRNA in DTC and showed a positive and significant correlation (Figure 3E; *P* = .014; *r*_s_ = 0.373; n = 43). The origin of this correlation in expression between CTTN and PBF was obscure as transfection of PBF failed to modulate protein levels of CTTN, and vice versa (Supplemental Figure 4). We therefore further evaluated CTTN and PBF expression in papillary thyroid cancer (PTC) by RNA-seq (TCGA). Consistent with our initial observations, we found a significant increase in CTTN expression (Figure 3F) in both nonmetastatic (N0; *P* = 5.6×10^−17^; n = 121) and metastatic PTC (N1; *P* = 7.5×10^−27^; n = 205) compared with normal thyroid (n = 59), as well as in different-sized tumors (Figure 3F), which was associated with subgroups of driver mutations (Supplemental Figure 5A). Similarly, PBF expression (Figure 3G) was induced in nonmetastatic (N0; *P* = 1.0×10^−7^; n = 121) and metastatic PTC (N1; *P* = 8.5×10^−13^; n = 205). Importantly, we again found a strong correlation between CTTN and PBF expression in PTC (Figure 3H; *P* = 4.7×10^−8^; *r*_s_ = 0.299; n = 326) and normal thyroid (Supplemental Figure 5B). CTTN and PBF expression in matched tumor/normal samples (n = 59) confirmed a strong correlation pattern (Supplemental Figure 5C; *P* = 2.74×10^−6^; *r*_s_ = 0.564).

Of particular significance, CTTN expression (Figure 3F) was higher in invasive metastatic PTC (N1; *P* = 3.6×10^−5^) than in nonmetastatic PTC (N0). TCGA clinical data also showed that higher CTTN expression was associated with stage IVA PTC (Figure 3I; *P* < .01), recurrence/progressed disease (Supplemental Figure 6; *P* < .029) and frequency of the BRAF mutation (Supplemental Figure 7; *P* < .001). Together our results demonstrate that CTTN is overexpressed in thyroid cancer, particularly in the most aggressive forms, and significantly correlates with PBF expression.

### CTTN mediates the influence of PBF on cell invasion

**Figure 4.**
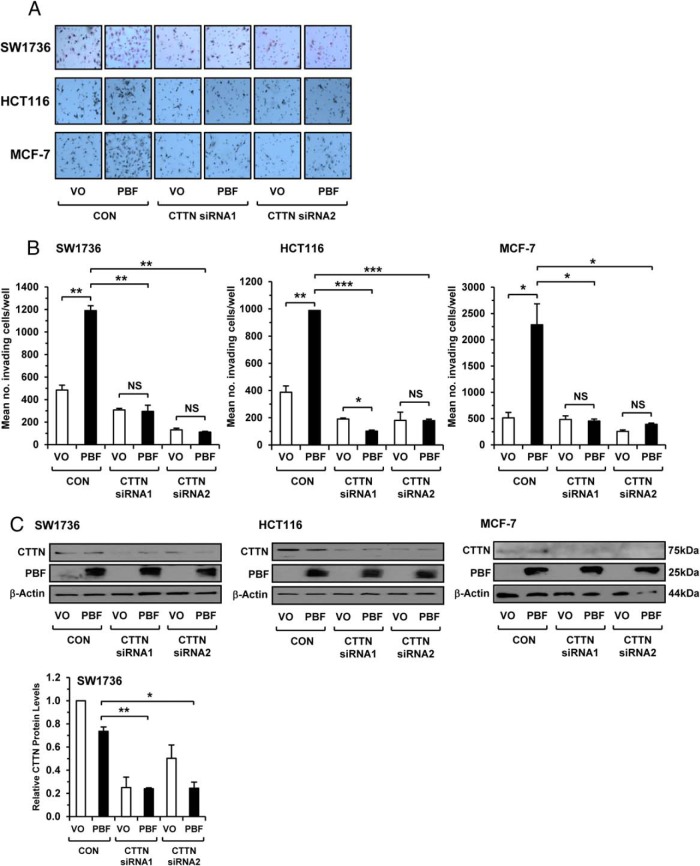
Depletion of CTTN ablates the ability of PBF to induce cellular invasion. A, Representative photomicrographs of 2D Boyden assay chamber cell invasion experiments in SW1736, HCT116, and MCF-7 cells transfected with VO or PBF, and then treated with specific siRNAs for CTTN (siRNA 1 and 2) or control siRNA (CON). B, Quantification of number of invading cells per well from 2D Boyden assay described in panel A. Results expressed as mean ± SEM from three independent experiments. *, *P* < .05; **, *P* < .01; ***, *P* < .001. C, Western blot analysis of CTTN and PBF-HA expression in response to siRNA treatments as outlined in panel A. Graph (bottom) shows quantification of CTTN protein levels relative to β-actin from siRNA experiments in SW1736 cells. Data presented as mean CTTN levels ± SEM from three independent experiments. *, *P* < .05; **, *P* < .01; ***, *P* < .001.

There have been inconsistent reports on the role of CTTN in cell motility, with a lack of involvement in the migration of CTTN-depleted mouse embryonic fibroblasts (25), as well as in 293T cells overexpressing CTTN (26). Given the interaction between CTTN and PBF, as well as significant correlations in thyroid cancer, we next depleted CTTN to determine the dependence of PBF on CTTN to promote cellular invasion. Critically, knockdown of CTTN by two different CTTN siRNAs (Figure 4, A and B) was associated with a significant reduction in the endogenous invasive capacity of SW1736 (*P* < .01), HCT116 (*P* < .001), and MCF-7 cells (*P* < .01) transfected with PBF. Of importance, the invasive capacity of CTTN-depleted cells transfected with PBF was equivalent (*P* = NS) or lower (*P* < .05) than vector-only (VO) control cells similarly depleted of CTTN (Figure 4B). Western blotting confirmed the reduction of endogenous CTTN protein by CTTN siRNA in all three cell lines, as well as exogenous PBF expression (Figure 4C). Together these results provide compelling evidence that the potent induction of cell invasion by PBF is driven in its entirety via CTTN.

### Phosphorylation modulates the functional interaction of PBF with CTTN

**Figure 5.**
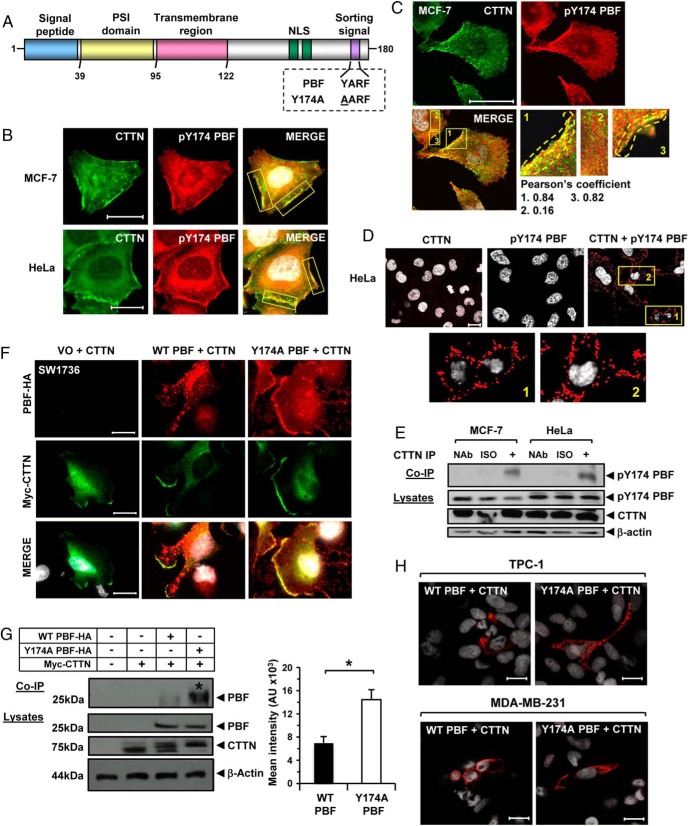
Involvement of Y174 in colocalization and binding of PBF with CTTN. A, Schematic of the protein domain structure of PBF showing the relative position of the YARF internalization motif. Boxed area highlights the alanine substitution at the Y174 residue in Y174A PBF. B, Representative immunofluorescence images showing subcellular colocalization (merge image, yellow) of endogenous CTTN (green) and endogenous pY174 PBF (red) in MCF-7 and HeLa cells. Intense “hot spots” of colocalization are outlined (yellow boxes). C, Confocal images showing colocalization (merge, yellow) between endogenous CTTN (green) and endogenous pY174 PBF (red) in MCF-7 cells. Framed areas in merge images are magnified and Pearson's colocalization coefficients are indicated. D, PLA assays showing specific interaction between endogenous CTTN and endogenous pY174 PBF in HeLa cells. Red fluorescent spots indicate specific interactions. Framed areas are magnified in lower panels. Scale bars, 10 μM. E, Representative Co-IP assays showing specific interaction between endogenous CTTN and endogenous pY174 PBF in MCF-7 and HeLa cells. F, Representative immunofluorescence images showing colocalization (merge, yellow) between myc-CTTN (green) and either WT PBF-HA (red) or Y174A PBF-HA (red) at the PM in SW1736 cells. Magnification, 100×. Scale bars, 20 μM. G, Co-IP assays showing increased binding (*) between myc-tagged CTTN and Y174A PBF vs WT PBF. Graph (right) shows quantification of PBF protein levels relative to β-actin from three independent experiments in HeLa cells. Data expressed as mean PBF levels ± SEM. *, *P* < .05. H, PLA assays showing specific interaction between Y174A PBF and CTTN in TPC-1 and MD-MBA-231 cells vs WT PBF. Magnification, 63×. Scale bars, 10 μM. White indicates DAPI nuclear staining in panels B–D, F, and H.

Phosphorylation at residue Y174 (Figure 5A; pY174) is critical to the interaction of PBF with thyroid-enriched NIS at the PM and its subsequent intracellular transport (23). We therefore investigated whether the phosphorylation status of PBF might influence its interaction with CTTN. To explore this we characterized endogenous CTTN and pY174 PBF, which demonstrated colocalization in MCF-7 and HeLa cells predominantly at the PM (Figure 5B), with significant overlap indicated by confocal microscopy (Figure 5C and Supplemental Figure 8; see Pearson's coefficient). Endogenous PLA (Figure 5D) and co-IP (Figure 5E) assays confirmed that pY174 PBF specifically associates with CTTN; thus physiological binding is plausible for residue pY174 in modulating the PBF:CTTN interaction at the PM.

The involvement of pY174 was next examined using PBF mutant Y174A, which disrupts phosphorylation at this residue and accumulates PBF at the PM by abolishing an internalization motif (23). Here we observed pronounced colocalization between Y174A PBF-HA and myc-CTTN at the PM, and reduced intracellular vesicular PBF compared with wild-type (WT) PBF (Figure 5F and Supplemental Figure 9). Interestingly, co-IP studies revealed that alanine substitution at residue Y174 was associated with markedly increased CTTN-binding (Figure 5G; >2-fold; *P* < .05). PLA assays in TPC-1 and MD-MBA-231 cells further suggested a strong interaction between Y174A PBF-HA and myc-CTTN (Figure 5H).

**Figure 6.**
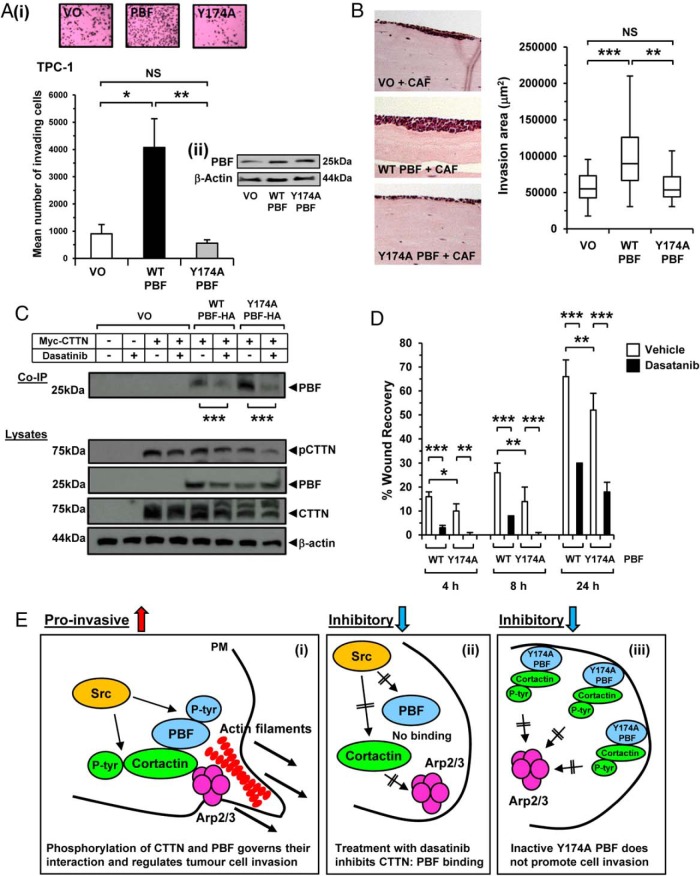
Phosphorylation is critical to the functional interaction of PBF with CTTN. A, Representative 2D Boyden assay chamber cell invasion assay from three independent experiments showing TPC-1 cells transfected with VO, WT PBF, or Y174A PBF; representative photomicrographs are shown (i), as well as Western blots to confirm transfection (ii). Results are expressed as mean ± SEM. *, *P* < .05; **, *P* < .01. B, Representative 3D organotypic invasion assays in MDA-MB-231 cells transfected with VO, WT PBF or Y174A PBF. Box-whisper plots (right) show quantification of invasion area of MDA-MB-2331 cells. Statistics analyzed using Mann-Whitney *U* test. **, *P* < .01; ***, *P* < .001. C, Co-IP assays in HeLa cells transfected with VO, WT PBF, Y174A PBF, or CTTN as indicated and then treated with 50nM dasatinib (+) or vehicle (−). D, Quantification of scratch wound assay in MCF-7 cells stably transfected with VO, WT PBF, or Y174A PBF. Cells were treated with 50nM dasatanib (black) or vehicle (white) and percent wound recovery determined at the indicated time point postrecovery (h). Results are expressed as mean ± SEM. *, *P* < .05; **, *P* < .01; ***, *P* < .001. E, Schematic representation summarizing the proposed mechanism of PBF binding to CTTN and regulating tumor cell invasion. Proinvasive: (i) Src phosphorylates PBF and CTTN thereby promoting their interaction and actin filament assembly via downstream partners such as Arp2/3. Inhibitory: (ii) Dasatinib inhibits tyrosine phosphorylation by Src and diminishes binding between PBF and CTTN. (iii) Ablating phosphorylation at residue Y174 inactivates PBF and impairs the induction of tumor cell invasion.

We next investigated whether the invasive properties of cells might be attenuated by modulating the phosphorylation status of PBF. Firstly, 2D Boyden chamber assays revealed that Y174A PBF was unable to stimulate cellular invasion in TPC-1 and MCF-7 cells (Figure 6A and Supplemental Figure 10; *P* = NS). These data were appraised in 3D organotypic assays in MDA-MB-231 cells, which confirmed the lack of cell invasion with Y174A PBF (Figure 6B). Both PBF and CTTN are phosphorylated by Src at residues Y174 and Y421, respectively (9, 23). Treatment with the Src kinase inhibitor dasatinib reduced CTTN phosphorylation by 30–50% and inhibited PBF:CTTN binding by approximately 50% (Figure 6C; *P* < .001). Y174A PBF again bound CTTN with higher avidity than WT PBF. Critically, dasatinib also significantly repressed binding between Y174A PBF and CTTN (Figure 6C; *P* < .001). In scratch wound assays, stable overexpression of Y174A PBF in MCF-7 cells was associated with significantly decreased wound healing compared with PBF (Figure 6D; *P* < .05). Importantly, dasatinib treatment inhibited wound healing of MCF-7 cells overexpressing WT PBF and Y174A PBF (Figure 6D and Supplemental Figure 11; *P* < .05). Subsequent studies demonstrated that the invasive properties of dasatinib-treated TPC-1 cells correlated with PBF phosphorylation status (Supplemental Figure 12).

Altogether these results demonstrate that tyrosine phosphorylation is critical to the functional interaction of PBF with CTTN in promoting tumor cell invasion. As CTTN and PBF colocalize at the leading edge of migrating cells, our data provides new mechanistic insights into blocking the movement of cancer cells by therapeutic targeting of this interaction. A schematic representation of our findings is presented in Figure 6E.

## Discussion

Differentiated thyroid cancer has a 5-year survival rate of 98.1%, but this decreases dramatically to 55.3% in patients with distant metastases (http://seer.cancer.gov/statfacts/html/thyro.html). The statistics are equally unpromising in metastatic breast cancer, where patients have a 5-year survival rate of just 26.3%. In general, given that it is the metastatic recurrence—and not the primary tumor—that ultimately proves fatal, the metastatic pathway requires continued investigation to establish new therapeutic targets. Through extensive mass spectrometry we identified the proto-oncogene PBF as a new binding partner of CTTN—a critical regulator of actin-cytoskeletal dynamics and associated with tumor aggressiveness. A clear interaction between PBF and CTTN at the cell periphery was evident by PLA analysis, which was further validated by co-IP and immunofluorescence assays. Importantly, functional CTTN expression was needed in order for PBF to induce invasion. Our current study therefore demonstrates that the proinvasive and promigratory action of PBF is contingent upon CTTN.

Expression of PBF has been circumstantially associated with metastasis in patients with breast (17) and thyroid cancer (16), and we recently reported that patients with colorectal cancer who demonstrate extramural vascular invasion have significantly higher PBF expression (18). However, these associations have not been appraised for causality, and the in vitro mechanisms by which PBF induces tumor cell invasion are unclear. In the present study we therefore utilized established thyroid, breast and colorectal models to address the mechanism by which PBF induces cell movement. A remarkable finding was that PBF consistently induced promigratory and proinvasive phenotypes across a panel of different cell types. Our results therefore imply that PBF may represent a novel marker of the ability of cells to escape tumors and invade, which provides further insight for the recent identification of PBF as a central driver gene in human cancers across multiple tumor types including thyroid (27).

CTTN expression has not been determined before in human thyroid tumors. In this study analysis of DTC specimens revealed that CTTN expression, whereas variable, was elevated in approximately two thirds of tumors. Importantly, analysis of a large clinically annotated TCGA dataset for PTC confirmed that CTTN was increased, especially in cases with lymph node metastasis. A significant positive association between PBF and CTTN mRNA was also evident in DTC specimens, as well as in TCGA, suggesting perhaps a commonality of gene regulation. This would not likely result from direct transcriptional regulation via either binding partner as neither CTTN nor PBF were capable of up-regulating the other. Previously, amplification of the gene that encodes CTTN has been reported in approximately 15% of primary metastatic breast carcinomas and approximately 30% of head and neck squamous cell carcinomas (28). Thus, the apparent induction of CTTN in DTC may be via gene amplification, or other gene regulatory mechanisms. Notwithstanding this, CTTN overexpression has been extensively linked to invasive cancers, including melanoma, colorectal cancer, and glioblastoma (8, 29, 30). Future studies will need to determine whether intratumoral heterogeneity stemming from genetic and nongenetic variability influences the expression and colocalization of CTTN and PBF.

Arguably the central challenge in human tumor therapy is to identify robust mechanisms of inhibiting cellular invasion and metastasis. To this end we appraised PBF as a gene which might represent a therapeutic paradigm in the attenuation of CTTN function. PBF shows the structural hallmarks of being an α-helical integral membrane protein (19), which continually shuttles to and from the PM (21). When we 'locked' PBF at the PM by disrupting its internalisation motif, we blocked the invasive capacity of cells in vitro, which was accompanied by significantly increased PBF:CTTN binding. CTTN regulates membrane trafficking as well as adhesion dynamics (8, 13), and PBF is intimately involved in both processes (21, 23, 31). Studies will need to address the potential cooperativity of PBF and CTTN in membrane trafficking and cytoplasmic localization, and whether the kinase pathways known to modulate aspects of PBF and CTTN function overlap in vitro and in vivo.

The principle sites of tyrosine phosphorylation of CTTN are Y421 and Y466, within the proline-rich domain (9). Src kinase phosphorylation at Y421 and Y466 is associated with lamaellipodial protrusions and recruitment of SH2 domain proteins such as Arg and NCK1, which links CTTN with N-WASP and WIP, and leads to enhanced activation of the Arp2/3 complex (32, 33). Of note, tyrosine phosphorylation of CTTN is also required for the endocytosis of several receptors (34). This is paralleled by one of the central functions of PBF, which is the regulation of the internalisation of PM proteins such as NIS (23, 31), and which is governed by tyrosine phosphorylation. We therefore attempted to define the effect of altered PBF phosphorylation on CTTN function via mutagenesis and dasatinib treatment. Key among dasatinib's established targets is Src, which phosphorylates both CTTN (9) and PBF (23). However, given that dasatinib has a range of targets and functions (35), disentangling the chronology and relative contribution of Src inhibition to altered CTTN and PBF function is challenging.

We therefore used a phospho-null PBF mutant (Y174A) in our dasatinib experiments to discriminate CTTN phosphorylation events from PBF phosphorylation. These data demonstrated that inhibiting Src reduced phosphorylation of CTTN at Y421 and decreased binding to PBF. Previous studies showed that Src phosphorylation of CTTN can increase its binding affinity to other binding partners such as Dynamin 2 by more than 3-fold (34). These findings are therefore supportive that tyrosine phosphorylation of CTTN promotes binding to PBF. Experiments to further define the role of Src phosphorylation at multiple sites in CTTN will be needed using phosphosite mutations at positions Y421 and Y466. From the PBF side, colocalization of pY174 PBF and CTTN was demonstrated at the PM, along with specific binding by co-IP analysis. Therefore, together with the lack of cellular invasion observed with Y174A PBF, these results suggest that phosphorylation of PBF at Y174 is required for a functional interaction with CTTN at the PM to mediate cell invasion.

Interestingly, Y174A PBF bound CTTN with a higher affinity than WT PBF. Our study inferred a role for PBF as a molecular scaffold or bridging molecule to promote the recruitment of CTTN with other CTTN-binding partners such as Src or the Arp2/3 complex and facilitate actin filament assembly. Y174A PBF might have therefore antagonized CTTN activity by blocking critical interactions with one or more recognized CTTN-binding partners due to its greater affinity for CTTN and increased retention at the PM. It is also likely that the altered properties of Y174A PBF would have delayed the dynamic regulation of the cortical actin cytoskeleton required for cell migration associated with multiple cycles of actin polymerization and depolymerization to generate the propulsive force needed for lamellipodia extension (10).

In summary, we present evidence that the proto-oncogene PBF is a novel interacting partner of the F-actin binding protein CTTN. PBF potently and consistently induces cell invasion and migration, which is dependent upon the presence of CTTN. As PBF and CTTN are induced in multiple tumor types, our study now identifies PBF as an undercharacterized target to block tumor cell movement. Ablating phosphorylation by mutation or treatment with a tyrosine kinase inhibitor modulates the interaction of PBF with CTTN and attenuates cell invasion. Hence, therapeutic strategies to manipulate the PBF:CTTN interaction need to be further appraised as promising new avenues in the treatment of endocrine tumor metastasis.
